# B cell treatment promotes a neuroprotective microenvironment after traumatic brain injury through reciprocal immunomodulation with infiltrating peripheral myeloid cells

**DOI:** 10.1186/s12974-023-02812-y

**Published:** 2023-05-31

**Authors:** Liam J. Dwyer, Saumya Maheshwari, Emily Levy, Mark C. Poznansky, Michael J. Whalen, Ruxandra F. Sîrbulescu

**Affiliations:** 1 Vaccine and Immunotherapy Center, Massachusetts General Hospital, Harvard Medical School, Boston, MA 02129 USA; 2 Neuroscience Center, Department of Pediatrics, Massachusetts General Hospital, Harvard Medical School, Boston, MA 02129 USA; 3 Department of Neurology, Massachusetts General Hospital, Harvard Medical School, Boston, MA 02114 USA

**Keywords:** Traumatic brain injury, B cells, Myeloid cells, Monocytes, Macrophages, Microglia, Immunomodulation

## Abstract

Traumatic brain injury (TBI) remains a major cause of death and severe disability worldwide. We found previously that treatment with exogenous naïve B cells was associated with structural and functional neuroprotection after TBI. Here, we used a mouse model of unilateral controlled cortical contusion TBI to investigate cellular mechanisms of immunomodulation associated with intraparenchymal delivery of mature naïve B lymphocytes at the time of injury. Exogenous B cells showed a complex time-dependent response in the injury microenvironment, including significantly increased expression of IL-10, IL-35, and TGFβ, but also IL-2, IL-6, and TNFα. After 10 days in situ, B cell subsets expressing IL-10 or TGFβ dominated. Immune infiltration into the injury predominantly comprised myeloid cells, and B cell treatment did not alter overall numbers of infiltrating cells. In the presence of B cells, significantly more infiltrating myeloid cells produced IL-10, TGFβ, and IL-35, and fewer produced TNFα, interferon-γ and IL-6 as compared to controls, up to 2 months post-TBI. B cell treatment significantly increased the proportion of CD206^+^ infiltrating monocytes/macrophages and reduced the relative proportion of activated microglia starting at 4 days and up to 2 months post-injury. Ablation of peripheral monocytes with clodronate liposomes showed that infiltrating peripheral monocytes/macrophages are required for inducing the regulatory phenotype in exogenous B cells. Reciprocally, B cells specifically reduced the expression of inflammatory cytokines in infiltrating Ly6C^+^ monocytes/macrophages. These data support the hypothesis that peripheral myeloid cells, particularly infiltrating monocyte/macrophages, are key mediators of the neuroprotective immunomodulatory effects observed after B cell treatment.

**Supplementary Information:**

The online version contains supplementary material available at 10.1186/s12974-023-02812-y.

## Background

Traumatic brain injury (TBI) remains a significant and acute medical need for which no therapeutic options are currently available. At a cellular level, many neurodegenerative effects associated with TBI can be traced to inflammatory responses after the initial insult [1]. The initial wave of trauma-related neuronal and glial necrotic cell death is followed by weeks and months of progressive secondary cell death [2]. This degenerative microenvironment activates both peripheral immune cells which infiltrate the brain in large numbers, and resident immune cells of the central nervous system (CNS), which consequently sustain a persistent inflammatory response [3, 4]. The neuroinflammatory response to acute CNS injury is multifactorial and highly dynamic, which may explain why therapeutic strategies that involve single-chemical immunomodulating drugs often fail to improve clinical outcomes. Direct dampening of the immune response after CNS injury, for example by using steroids such as methylprednisolone does not improve neurological outcomes [5], and may even be detrimental [6]. By contrast, strategies that recognize and address the complex microenvironment of the CNS injury may be more promising. Cell-based therapies are versatile and potentially multifunctional, capable of immunomodulation in the context of complex and dynamic pathologies that engage multiple different interactions between cell types [7].

Our previous work has demonstrated that exogenous mature naïve B220^+^/CD19^+^/IgM^+^/IgD^+^ B cells are potent modulators of inflammatory responses, and that B cell treatment was associated with structural and functional neuroprotection after TBI [8, 9]. In a murine TBI model using controlled cortical impact (CCI) injury, a single intraparenchymal injection of purified splenic B cells at the time of injury was shown to significantly improve cognitive functional recovery after CCI in a variety of neurobehavioral paradigms, and to reduce brain tissue loss by 40–60% as compared to injured groups treated with saline or equivalent numbers of splenic T cells [8]. Naïve B cells placed in injured microenvironments become activated via Toll-like receptor (TLR)—MyD88-dependent signaling pathways and can generate a variety of immunoregulatory cytokines that lead to accelerated or improved tissue repair [9]. The anti-inflammatory cytokines IL-10 and IL-35 may be critical B cell-derived effector molecules that modulate healing in this context, since loss of IL-10 significantly reduced the neuroprotective effect of B cells [9]. While the regulatory and protective effects of B cells have been reproducibly described in a variety of tissues and model systems [9–13], the cellular mechanisms which underlie these effects are still poorly understood.

The structural and functional benefits of B cell administration far outlast the limited lifespan of the exogenous B cells in situ [8], suggesting that other cell types within the injured microenvironment may act as relays or secondary effectors of the immunomodulation initiated by the B cell application. Here, we address this hypothesis in a stepwise manner by investigating the effect of B cell therapy on infiltrating and resident immune and inflammatory cells after TBI, with a specific focus on myeloid populations. After CNS trauma, myeloid cells are the numerically dominant immune cell population infiltrating into the site of injury through the permeabilized blood–brain barrier [1, 2, 14]. In particular, the infiltration of monocytes into the brain parenchyma after TBI has been associated with a detrimental effect on neuronal survival and functional recovery [15]. There is strong evidence that B cells can closely interact with macrophages in the context of the adaptive immune response as well as in autoimmunity [16–19]. Previous studies have also found that intraparenchymal B cell treatment can significantly reduce the activation of resident microglia as late as 35 days after CCI [8], supporting a potential regulatory interaction with myeloid cell populations. In this study, we examined the response of exogenous B cells to the microenvironment of a CCI injury and their reciprocal regulatory relationships with infiltrating peripheral myeloid cells. We find evidence that supports the view that infiltrating peripheral monocytes/macrophages are required for the regulatory activation of the transplanted B cells, and are in turn subject to the modulatory influence of the exogenous B cells, mediating their functional neuroprotective effects.

## Materials and methods

### Animals

Studies were conducted in 8- to 12-week-old male C57Bl6/J mice (Jackson Laboratories). Male C57Bl6/J wild type (WT), B6.SJL-Ptprc^a^Pepc^b^/BoyJ (CD45.1^+^), or B6.129P2-Il10^tm1Cgn^/J (IL-10^−/−^) mice were used as isogeneic donors for B cell isolation. Animals were maintained under standard laboratory care conditions, at temperatures ranging between 20 and 23 °C, under a 12 h:12 h light:dark cycle, with ad libitum access to food and acidified water. All animal procedures were performed following the Public Health Service Policy on Humane Care of Laboratory Animals and were approved by the Institutional Animal Care and Use Committee of Massachusetts General Hospital. Animals were matched for age and randomly assigned to experimental conditions. To avoid bias, animals from different treatment arms were always co-housed. All efforts were made to reduce the number of animals used and to minimize animal suffering.

### B cell isolation

B cell isolation was performed using negative immunomagnetic selection as described previously [12]. Mouse spleens were collected in ice-cold phosphate-buffered saline (PBS) containing 2% fetal bovine serum (FBS) and 1 mM ethylenediaminetetraacetic acid (EDTA). Spleens were dissociated mechanically through a 40-µm cell strainer and the splenocyte suspension was processed for immunomagnetic negative B cell selection, using commercially available cell isolation kits (STEMCELL Technologies) according to the manufacturer’s instructions. The purity of the B cell isolate was over 98% mature naïve CD45R^+^/CD19^+^ B lymphocytes, as validated by flow cytometry analysis [12]. Purified B lymphocytes were re-suspended in sterile PBS at a concentration of 2.5 × 10^6^ cells/µl.

### Controlled cortical impact

CCI was performed as described previously [8]. Anesthesia was induced with 3.5% isoflurane (Baxter, Deerfield, IL) for 90 s in a mixture of 70% N_2_O and 30% O_2_ using a Fluotec 3 vaporizer (Colonial Medical, Windham, NH) and maintained with 3% isoflurane. Mice were placed in a stereotactic frame and a 5-mm craniotomy was made over the left parieto-temporal cortex. B cells suspended in sterile saline or only sterile saline (control) were injected intraparenchymally at − 1 mm from bregma on the anterior/posterior axis, + 2 mm medial/lateral, at a depth of 3 mm through the left parietal cortex. A total volume of 4 µl containing 2.5 × 10^6^ B cells/µl was injected immediately before CCI using a 10-µl Hamilton syringe with a 26 s gauge blunt-tip needle (Hamilton Company, Franklin, MA). Control animals received saline only. Mice were randomly assigned to the selected treatment or control condition. Intraparenchymal injections were performed immediately before CCI to ensure correct and consistent injection of cells while the brain structure was intact. Mice were immediately thereafter subjected to CCI using a pneumatic cylinder with a 3-mm flat-tip impounder, at a velocity of 6 m/s, a depth of 0.6 mm and a 100-ms impact duration. Sham-injured mice underwent anesthesia, craniotomy, and intraparenchymal injection with equal numbers of B cells or saline, but no CCI injury. The craniotomy was left open, and the skin was closed over the skull using 5-0 nylon sutures (Ethicon, Cornelia, GA).

### Sample collection and processing

At specified intervals after CCI and treatment (18 h, 48 h, 96 h, 10 d, or 2 mo), the mice were deeply anesthetized with ketamine (100 mg/kg) and xylazine (10 mg/kg), perfused transcardially with 10–15 ml of heparinized PBS to remove blood, and decapitated. For experiments that involved the evaluation of intracellular cytokines, 4–5 h prior to sample collection, animals were anesthetized with 3% isoflurane in O_2_ and received an intravenous injection of 250 μg Brefeldin A, a macrolide that disrupts protein secretion in eukaryotic cells, to promote the accumulation of cytokines inside cells [20].

For histology, the brains were rapidly extracted on ice, frozen in liquid nitrogen vapor, and stored at − 80 °C. Before cryosectioning, the brains were embedded in M-1 embedding matrix (Thermo Fisher Scientific, Waltham, MA), and sectioned coronally at a thickness of 14 µm using a cryostat. Serial sections were collected at 150 μm intervals along the rostro-caudal axis in the area of the injury, and thaw-mounted onto SuperFrost Plus Gold slides (Fisher Scientific, Waltham, MA).

For flow cytometry analysis, the brains were rapidly extracted on ice and a 6-mm biopsy punch was used to excise a standardized tissue biopsy of approximately 115 mm^3^ comprising the ipsilateral injury site and an equivalent sample from the uninjured contralateral hemisphere. Brain tissue samples were first mechanically triturated and then enzymatically dissociated for 25 min at 37 °C, using a papain-based method according to the manufacturer’s specifications (Miltenyi Biotec, Bergisch Gladbach, Germany). Prior to immunolabeling, myelin was removed from cell suspensions using commercially available myelin depletion beads (Miltenyi Biotec).

### Flow cytometry

To evaluate viability, the cell suspensions were washed and resuspended in PBS and stained using a Zombie UV fixable dye (Biolegend) for 30 min. The cells were then washed and resuspended in PBS containing 1% FBS, 0.01% sodium azide (RICCA Chemical) and 5% FcR blocking reagent (Miltenyi Biotec) for 10 min. Blocked cells were then incubated for 30 min with the following fluorophore-conjugated primary surface antibodies: Brilliant Ultraviolet 395-conjugated Armenian hamster anti-mouse CD69 (clone H1.2F3) (BD Biosciences, San Jose, CA), Brilliant Ultraviolet 737-conjugated rat anti-mouse CD11b (clone M1/70) (BD Biosciences, San Jose, CA), Brilliant Violet 421- or Brilliant Violet 785-conjugated rat anti-mouse CD19 (clone 6D5), Brilliant Violet 421-conjugated mouse anti-mouse CX3CR1 (clone SA011F11) (Biolegend), Brilliant Violet 510-conjugated rat anti-mouse CD8α (clone 53–6.7), Brilliant Violet 510-conjugated rat anti-mouse CD86 (clone GL-1) (Biolegend), Brilliant Violet 605-conjugated rat anti-mouse Ly6C (clone HK1.4), Brilliant Violet 605-conjugated rat anti-mouse CD274(PD-L1) (clone 10F.9G2) (Biolegend), Brilliant Violet 650-conjugated rat anti-mouse Ly-6C (clone HK1.4) (Biolegend), Brilliant Violet 711-conjugated rat anti-mouse CD3 (clone 17A2), Brilliant Violet 711-conjugated rat anti-mouse CD68 (clone FA-11) (Biolegend), Brilliant Violet 785-conjugated Armenian hamster anti-mouse CD11c (clone N418) (Biolegend), Alexa Fluor^®^ 488-conjugated mouse anti-mouse CD45.1 (clone A20), PerCP/Cy5.5 conjugated rat anti-mouse CD4 (clone GK1.5), Alexa Fluor 488-conjugated rat anti-mouse CD74 (clone In1/CD74) (Biolegend), PerCP/Cyanine5.5-conjugated rat anti-mouse CD170(Siglec-F) (clone S17007L) (Biolegend), PE-CF594-conjugated rat anti-mouse Ly6G (clone 1A8), PE-conjugated rat anti-mouse CD39 (clone Duha59) (Biolegend), PE/Dazzle 594-conjugated rat anti-mouse CD172a(SIRPa) (clone P84) (Biolegend), PE/Cy7-conjugated rat anti-mouse CD11b (clone M1/70), PE/Cy7-conjugated rat anti-mouse CD163 (clone S15049I) (Biolegend), Alexa Fluor^®^ 647-conjugated rat anti-mouse CD206 (clone C068C2), Alexa Fluor^®^ 700-conjugated rat anti-mouse/human CD45R/B220 (clone RA3-6B2), Alexa Fluor 700-conjugated rat anti-mouse Ly-6G (clone 1A8) (Biolegend), APC/Cy7-conjugated rat anti-mouse CD45 (clone 30-F11) (all from Biolegend). Surface-stained cells were washed and resuspended in fixation buffer (Biolegend) for 20 min, followed by permeabilization wash buffer (1X) (Biolegend). Permeabilized cells were incubated for 30 min with the following intracellular antibodies: PE-conjugated Armenian hamster anti-mouse IL-1α (clone ALF-161), Brilliant Violet 421-conjugated mouse anti-mouse TGF-β1 (clone TW7-16B4), Brilliant Violet 510-conjugated rat anti-mouse IFNγ (clone XMG1.2), Brilliant Violet 711- or Brilliant Violet 785-conjugated rat anti-mouse TNFα (clone MP6-XT22), PerCP/Cy5.5 conjugated rat anti-mouse IL-2 (clone JES6-5H4), PE-conjugated rat anti-mouse IL-27/35 (clone 355022) (R&D Systems, MN), PE/Cy7-conjugated rat anti-mouse IL-10 (clone JES5-16E3), APC-conjugated rat anti-mouse IL-6 (clone MP5-20F3). All incubation steps were performed at 4 °C, protected from light. Cells were analyzed on an LSR Fortessa X-20 flow cytometer (BD Biosciences) equipped with 355 nm, 405 nm, 488 nm, 561 nm and 640 nm lasers, using BD FACSDiva™ software. At least 800,000 events were collected from each sample for analysis. Data were analyzed using FlowJo software, version 10.8.1 (TreeStar, Inc.).

### Immunohistochemistry

After washing with PBS, the sections were permeabilized and blocked with 5% bovine serum albumin, 5% FBS, and 0.3% Triton X-100 for 1 h at room temperature. Sections were then incubated overnight at 4 °C with the following primary antibodies diluted in blocking solution: Alexa Fluor^®^ 488-conjugated mouse anti-mouse CD45.1 (clone A20), PE-conjugated rat anti-mouse CD11b (clone M1/70), Alexa Fluor^®^ 647-conjugated rat anti-mouse CD22 (clone OX-97). Unbound primary antibody was removed by three rinses in PBS. Sections were counterstained by incubation with 2 μg/ml of 4′, 6-diamidino-2-phenylindoledihydrochloride (DAPI; Sigma Aldrich).

### Confocal microscopy

Stained tissue sections were imaged using a Zeiss LSM 710 laser scanning microscope (Carl Zeiss) equipped with 10×, 20×, 40×, 63×, objectives. Confocal images were collected using Zen software (Carl Zeiss) at a resolution of 0.23–0.71 µm/pixel.

### Systemic depletion of monocytes/macrophages

For monocyte/macrophage depletion, recipient mice were injected intravenously with 10 µl/g body weight of clodronate-loaded liposome suspension (Liposoma, Amsterdam, Netherlands). Control animals received the same dosage of saline-loaded (control) liposomes. Monocyte/macrophage depletion was verified by flow cytometry analysis in multiple tissues.

### Behavioral testing

Behavioral testing was conducted during the light phase of the circadian cycle, as previously described [9]. Mice were allowed at least 30 min to acclimate to the testing environment. Rotarod: mice were placed on an automated rotarod apparatus (Harvard Apparatus, Holliston, MA), which accelerated from 4 to 40 rotations/min over 120 s. Maximum trial duration was 300 s, or until the mouse fell off the rotarod. The average latency to drop and the average rotations/min speed attained over three trials were recorded for each day of testing. The apparatus was cleaned and disinfected between trials with a hydrogen peroxide-based solution (Peroxigard, ON, Canada). Y-maze spontaneous alternation test: the Y-maze test was conducted in an apparatus constructed of white opaque acrylic, consisting of three 40-cm-long arms joined at 120-degree angles, with a wall height of 15 cm. Each arm was labeled with a different contrasting visual cue (black-on-white square, circle, triangle). Mice were placed in the center of the apparatus and allowed to explore the maze for 5 min. Their movements were recorded using a webcam positioned directly overhead and ANY-maze software (Stoelting Co., Wood Dale, IL). Normal exploratory behavior in rodents involves a preference to enter a less recently visited arm of the maze (spontaneous alternation). An alternation percentage was calculated by dividing the number of three successive choices that included one instance of each arm by the total number of arm entries minus one (i.e., total opportunities for alternation). The apparatus was cleaned and disinfected between trials with a peroxide-based solution (Peroxigard, ON, Canada).

### Statistics

Sample sizes were determined based on previously published estimations for effect size and power for known paradigms [8, 9] or otherwise determined as the minimal number of animals required for pairwise testing (e.g., validation testing for established paradigms, such as clodronate-based depletion of macrophages). Statistical analyses were performed using GraphPad Prism 9 (GraphPad Software). Prior to statistical testing, datasets were assessed for normal distribution using the D'Agostino and Pearson normality test. All reported descriptive statistics are estimated marginal means ± standard error of the mean (SEM). P values < 0.05 were considered statistically significant.

## Results

### Exogenous B cells exhibit a complex time-dependent immunomodulatory response to the microenvironment of the injured brain

**Fig. 1:**
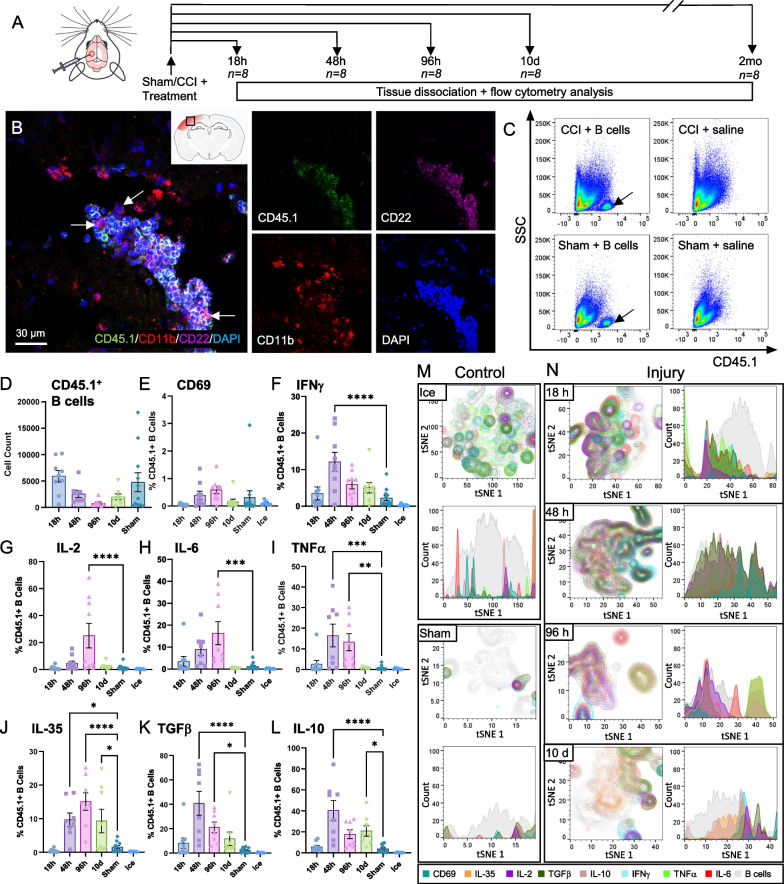
Time-dependent response of exogenous mature naïve B cells at the site of CCI injury. **A** Schematic representation of injury and treatment paradigm. B cells are injected intraparenchymally at the time of CCI, and both injured (ipsilateral) and contralateral brain hemispheres are collected for analysis at determined intervals. **B** At 18 h after CCI, exogenous CD45.1^+^ CD22^+^ B cells injected at the time of lesion remain aggregated at the site of tissue injury, while infiltrating CD11b^+^ myeloid cells surround and intersperse with the B cells (arrows). **C** Exogenous CD45.1^+^ B cells can be readily identified in flow cytometry analysis of dissociated tissue from treated mice, in both injured and sham-injured brain hemispheres. By contrast, saline-treated controls show minimal B cell infiltration. Sample results are shown at 18 h post-injury and treatment. **D** The number of B cells at the injury site decreases steadily over time, and by 2 months post-CCI and treatment, no B cells could be observed in the biopsies collected from the injury site. **E–L** Increasing proportions of the retrieved populations of B cells injected into the injured brain microenvironment became activated over time, producing both pro- and anti-inflammatory cytokines. Note that while inflammatory cytokines such as IL-6, IFNγ, or TNFα peak early after CCI injury, a large proportion of the exogenous B cells continue to produce regulatory cytokines, including IL-35, IL-10, and TGFβ, persistently, at least up to 10 days after administration. Statistical significance was assessed by one-way ANOVA, followed by Dunnett's multiple comparisons test against the sham-injury control. *p < 0.05; **p < 0.01; ***p < 0.001; ****p < 0.0001. **M** Minimal activation and cytokine production was observed in the control conditions, when B cells were maintained on ice or exposed to an uninjured brain microenvironment. **N** By contrast, the majority of B cells injected at the lesion site begin producing a combination of cytokines by 18 h, peaking at 48–96 h post-injury. tSNE analysis indicated that there was a substantial overlap between the source cells for a variety of pro- and anti-inflammatory cytokines. By 4 days and even more so at 10 days post-application, subsets of B cells that produce exclusively regulatory cytokines, IL-10 and IL-35, become apparent and dominate the B cell population within the injury. In histograms, tSNE counts are normalized to mode

To assess the cellular response dynamics in the microenvironment of the injured brain, purified splenic B cells were injected into the brain parenchyma immediately prior to CCI injury. This procedure ensured that a bolus of B cells was present at the core of the impacted brain tissue. Samples were then collected at acute, subacute, and chronic time intervals—up to 2 months after injury (Fig. 1A). Histological examination of tissue sections confirmed that the applied B cells remained localized at the injection site, as previously observed in this model [8]. While in other models of brain injury B cells have been reported to adopt a CD11b^+^ activated phenotype that was associated with neuroinflammation [21], limited CD11b expression was observed in the exogenous CD45.1^+^CD22^+^ B cells in the current study. Instead, CD11b was present on peripheral monocytes/macrophages, which infiltrated the injured tissue as early as 18 h post-injury (Fig. 1B). Flow cytometry analysis of dissociated tissue from injured or sham-injured brain hemispheres confirmed that the exogenous B cells administered therapeutically can be identified with high specificity and were only present in cell-treated animals and not in saline-treated controls (Fig. 1C). To ensure that cytokines accumulate within cells and are thus detectable by flow cytometry, all animals received intravenous injections of brefeldin A, a macrolide that disrupts protein secretion in eukaryotic cells [20], four hours before sample collection. Analysis of the cytokine response in the exogenous B cells after exposure to brefeldin A in vivo showed that the cells undergo an initial lag period of activation of approximately 24 h, followed by a maximal response at 48-96 h post-injection (Fig. 1E–L, Additional file 1: Fig. S1). At the latter time points, 40–60% of the exogenous B cells were positive for both pro- and anti-inflammatory cytokines. This complex generalized response of exogenous B cells to the injured microenvironment has been described previously in wound healing as *pligodraxis* [9]. Interestingly, tSNE analysis of the data for each cytokine showed that the same B cells were producing both regulatory cytokines such as TGFβ and classical proinflammatory factors such as IFNγ or TNFα (Fig. 1M, N). At later time points, 4–10 days, the regulatory phenotype becomes dominant, with the exogenous B cells maintaining only the expression of IL-10 and IL-35 significantly above baseline. This effect was reflected in the emergence of large distinct subsets of regulatory B cells that only express these cytokines, while other, diminished subsets still express a mix of pro- and anti-inflammatory cytokines (Fig. 1N). At 2 months post-CCI and treatment, the numbers of exogenous B cells were very low, not exceeding baseline, making it unlikely that the transplanted cells would still exert a direct effect long-term.

### Intraparenchymal B cell application at the time of injury does not alter the proportion of infiltrating and resident myeloid immune cells

**Fig. 2:**
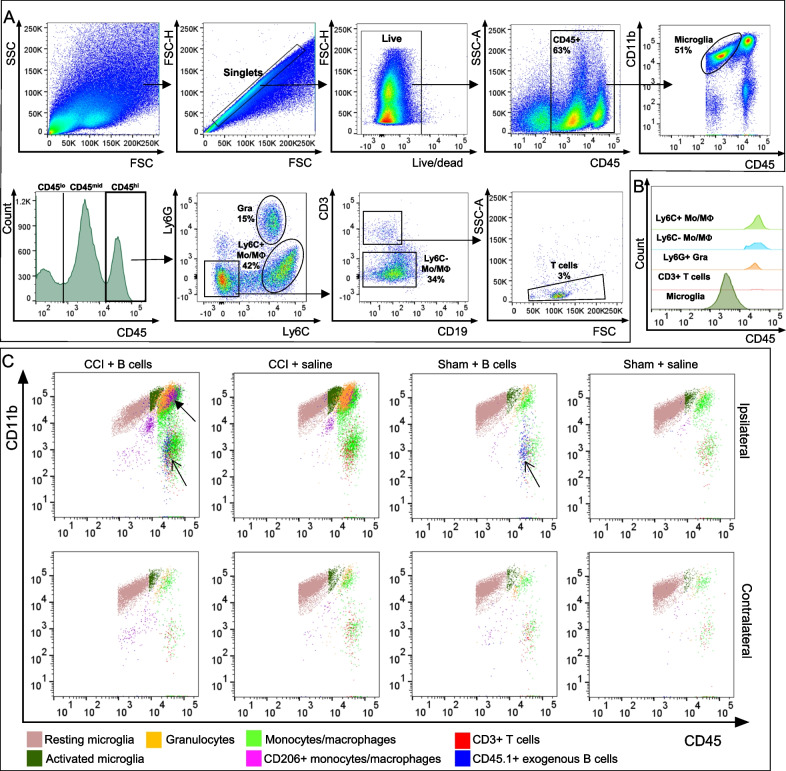
Multiplexed flow cytometry identification of cell populations in the CCI microenvironment. **A** Gating strategy for identifying resident and infiltrating immune cells after CCI. **B** Relative proportions of resident and infiltrating immune cells in the injured brain. In addition to the CNS-resident microglia, most infiltrating leukocytes are also of myeloid origin. Example shown at 96 h post-injury. **C** Immune cell populations in brain tissue from injured and sham-injured ipsilateral and contralateral hemispheres were analyzed. Gated populations were overlayed to show differential expression of CD45 and CD11b. Exogenous CD45^hi^ CD11b^−^ B cells can be found in treated injured and sham-injured animals (open arrows). A population of CD206^+^ monocytes/macrophages appeared distinctly enriched in the injured brain, particularly in animals treated with B cells (arrow)

**Fig. 3:**
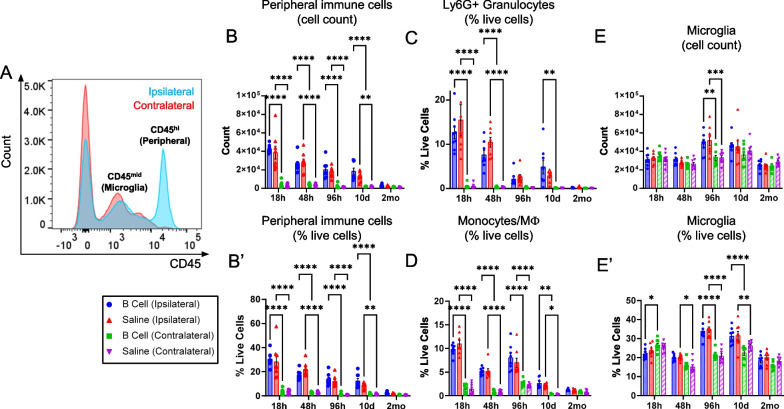
CCI injury is associated with substantial infiltration of CD45^hi^ immune cells into the brain. **A** Histogram illustrating the proportion of infiltrating (CD45^hi^) and resident (CD45^mid^) immune cells as well as non-immune cells (CD45^−^) in the injured (ipsilateral) or non-injured (contralateral) brain hemispheres in a representative sample at 18 h post-CCI. **B**, **B′**. Total numbers and relative proportions of infiltrating CD45^hi^ immune cells are highly increased by the injury, peak at 18 h post-CCI, and gradually decrease over time, returning to baseline by 2 months post-CCI. No effect of B cell treatment can be observed as compared to saline-treated controls. Similarly, B cell application did not change the proportion of infiltrating neutrophils **C** or monocytes/macrophages **D**, which represent the majority of the immune infiltrate at the lesion site. **E**, **E′**. Quantitative analysis of CNS-resident immune cells also showed that the total numbers and relative proportion of microglia out of the total live cells only show injury-dependent significant changes. An initial decrease in cell numbers at 18 h was followed by a significant injury-associated increase at 48–10 days post-CCI. There was no significant difference between the injured and non-injured hemispheres by 2 months post-injury. Statistical significance was determined using two-way ANOVA, followed by Tukey's multiple comparisons test. *p < 0.05; **p < 0.01; ***p < 0.001; ****p < 0.0001

We hypothesized that the introduction of mature naïve B cells into the injured brain parenchyma may directly change the dynamics of peripheral immune cell infiltration or the relative proportions of infiltrating immune cell subsets. Using multiplexed flow cytometry in brain tissue biopsies standardized for volume, we examined the composition of peripheral CD45^hi^ and resident CD45^mid^ immune components of the ipsilateral and contralateral brain hemispheres after CCI (Fig. 2). The CD45^hi^ immune infiltrate comprised primarily myeloid cells, granulocytes and monocyte/macrophages, with only approximately 3% of infiltrating cells being CD3^+^ T cells (Fig. 2B). As expected, immune infiltration as well as microglial activation was primarily associated with the injury. In this paradigm, contralateral hemispheres of both injured and sham-injured animals similarly showed minimal levels of peripheral immune cell infiltration (Figs. 2C, 3A). Both total cell numbers and relative proportions of infiltrating peripheral CD45^hi^ immune cells are highly increased by the injury, with peak values reached at 18 h post-CCI, followed by a gradual decrease over time. By 2 months post-CCI, cellular infiltrate is not significantly different from baseline levels (Fig. 3B, B′). Administration of B cells at the time of injury was not associated with a change in the relative proportion of infiltrating CD45^hi^ immune cells. More detailed examination of major cellular subsets showed that the same was true for the relative numbers of infiltrating neutrophils (Fig. 3C) and monocytes/macrophages (Fig. 3D), which were not altered by the B cell treatment. Unlike infiltrating CD45^hi^ immune cells, CD45^mid^ microglia are present in situ at the time of CCI and respond to injury by proliferating and migrating towards the injury site [1], resulting in a significant increase in cell numbers in the injured hemisphere at 4–10 days post-CCI. This response was not altered by the application of B cells at the injury site (Fig. 3E, E′).

### Treatment with exogenous B cells induces an early and persistent anti-inflammatory phenotype in infiltrating and CNS-resident immune cells

**Fig. 4:**
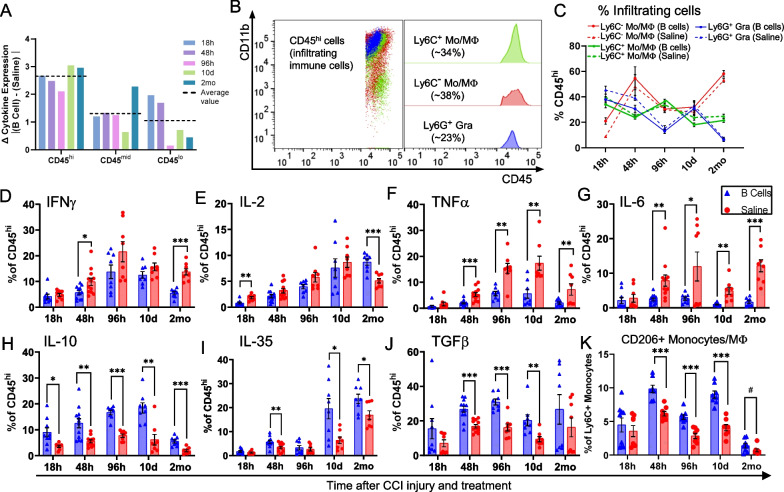
B cell application modulates the functional phenotype of infiltrating myeloid immune cells at the site of injury. **A** Analysis of the differential aggregate cytokine response in cells isolated from the brain of saline-treated and B cell-treated animals showed that the largest overall response at all analyzed time points was observed in the infiltrating CD45^hi^ immune and inflammatory cells. **B** Monocytes/macrophages and neutrophils comprise approximately 95% of the CD45^hi^ infiltrating immune and inflammatory cells. **C** Temporal dynamics of the relative proportions of CD45^hi^ cell subsets. In both B cell-treated (*continuous lines*) and saline-treated (*dashed lines*) animals, granulocytes declined steadily over time, Ly6C^+^ monocytes/macrophages showed a slight decline and Ly6C^−^ monocyte/macrophages increased substantially up to 2 months post-injury. **D–J** Analysis of cytokine production in the CD45^hi^ infiltrating immune cell populations showed a significant impact of B cell treatment, with a complex pattern of immunomodulation. Overall, the proportion of CD45^hi^ cells expressing high levels of canonical pro-inflammatory cytokines IFNγ, TNFα, and IL-6 was lower in animals treated with B cells (**D**, **F**, **G**). Conversely, more CD45^hi^ cells expressed high levels of regulatory cytokines IL-10, IL-35, and TGFβ (**H**, **I**, **J**). These regulatory patterns were observed into the chronic stages, at 2 months post-CCI. **K** The proportion of Ly6C^+^ inflammatory monocytes/ macrophages expressing the M2-associated marker CD206 was significantly increased in B cell-treated animals up to 10 days post-CCI and was still elevated, though trending to baseline levels at 2 months. Overall effects were assessed using 2-way ANOVA, and statistical significance at each timepoint was assessed by multiple two-tailed unpaired t-tests for each individual marker and time point and corrected for multiple comparisons using the Holm-Šídák method. ^#^*p* < 0.1; *p < 0.05; **p < 0.01; ***p < 0.001; ****p < 0.0001

To examine which cell types respond functionally to the introduction of exogenous B cells into the CCI microenvironment, we first used multiplexed flow cytometry in standardized tissue biopsies to evaluate the production of seven key cytokines in three major cell subsets—CD45^hi^ infiltrating immune cells, CD45^mid^ microglia, and CD45^neg^ non-immune cells. When all changes in cytokine production were considered in aggregate, the largest overall response to the presence on B cells was observed in the CD45^hi^ infiltrating immune cells at all time points examined (Fig. 4A). In immune and non-immune resident cells, the response was overall more limited, except at 2 months post-injury, when the initial B cell treatment induced a large overall differential response in CD45^mid^ microglia. We thus focused our initial analysis on the CD45^hi^ infiltrating immune cell compartment, which predominantly comprised Ly6G^+^ granulocytes, Ly6C^+^ classical monocytes/macrophages, and Ly6C^−^ non-classical monocytes /macrophages. Together, these three myeloid subsets represented approximately 95% of the infiltrating CD45^hi^ cells at all time points examined (Fig. 4C). Within the CD45^hi^ compartment, granulocytes represented approximately 23% of the CD45^hi^ subset, declining over time post-injury, while Ly6C^+^ and Ly6C^−^ monocytes/macrophages represented on average 34% and 38%, respectively, with Ly6C^−^ subsets dominating at later time points after injury (Fig. 4C). In aggregate, the CD45^hi^ infiltrating immune cell compartment was strongly modulated by the presence of exogenous B cells at all time points. Main effects of B cell treatment were observed for classical pro-inflammatory cytokines IFNγ (F_(1, 79)_ = 19.87, *p* < 0.0001), TNFα (F_(1, 79)_ = 53.44, *p* < 0.0001), and IL-6 (F_(1, 79)_ = 34.92, *p* < 0.0001), which were expressed in a significantly lower percentage of the CD45^hi^ population, while regulatory cytokines IL-10 (F_(1, 79)_ = 75.45, *p* < 0.0001), IL-35 (F_(1, 79)_ = 22.62, *p* < 0.0001), and TGFβ (F_(1, 79)_ = 20.28, *p* < 0.0001) were highly expressed in a significantly larger proportion of the CD45^hi^ infiltrating immune cells (Fig. 4D–J). Moreover, among Ly6C^+^ monocytes/macrophages, considered to have a classical pro-inflammatory phenotype [22] a significantly higher proportion expressed CD206, a marker of alternative, regulatory macrophage activation [23] when B cells were administered at the lesion site (Fig. 4K), and this trend was maintained at chronic time points post-CCI (F_(1, 71)_ = 51.18, *p* < 0.0001).

**Fig. 5:**
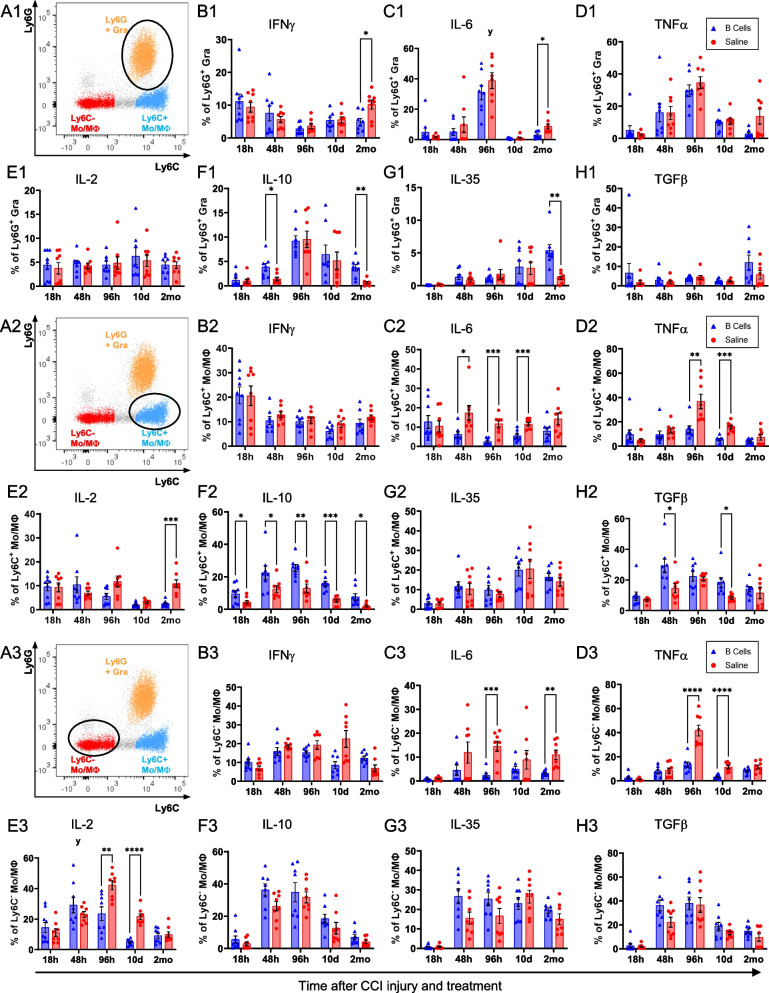
Immunomodulatory effect of B cell application within the main subsets of infiltrating myeloid immune cells at the site of injury. **A1**–**H1** Ly6G^+^ granulocytes showed time-dependent cytokine dynamics mostly driven by injury progression, with only modest differential response to the presence of B cells. Interestingly, a regulatory effect in B cell-treated animals, associated with increased production of IL-10 and IL-35 and a reduction in IFNγ and IL-6 was observed mostly at the chronic, 2-month time point. **A2**–**H2** Ly6C^+^ monocytes/macrophages showed the strongest overall differential response to B cell treatment, with a significant reduction in the numbers of cells producing proinflammatory cytokines such as TNFα, IL-2, and IL-6, and conversely an increase in cells that produced IL-10 and TGFβ. **A3**–**H3** Ly6C^−^ non-classical monocytes/macrophages were also strongly regulated by the application of B cells at the injury site, primarily by reducing the numbers of cells that produced proinflammatory cytokines TNFα, IL-2, and IL-6. Statistical significance was assessed by multiple two-tailed unpaired t-tests for each individual marker and time point and corrected for multiple comparisons using the Holm-Šídák method *p < 0.05; **p < 0.01; ***p < 0.001; ****p < 0.0001

In order to deconvolve the B cell-dependent response of the CD45^hi^ infiltrating cells, we next examined the same set of cytokine responses separately, in each of the major myeloid subsets that comprise this population (Fig. 5). In the Ly6G^+^ granulocyte subset, cytokine expression was only modestly impacted by the administration of B cells (Fig. 5A1–H1). Most of the changes in cytokine response were time-dependent, and likely associated with injury progression, except at 2 months post-CCI, when a regulatory effect was observed in B cell-treated animals, with more granulocytes producing IL-10 and IL-35 and a reduction in cell producing IFNγ and IL-6. Of note, the absolute number of granulocytes still present in the injured brain parenchyma at 2 months post-CCI was very low, totaling only approximately 200–400 cells per injury site (Fig. 3C). By contrast, monocytes/macrophages appeared to drive the significant responses to B cell administration detected in the overall CD45^hi^ infiltrating population. Ly6C^+^ classical monocytes/macrophages showed the most complex overall differential response to B cell treatment, with a significant reduction in the percentage of cells producing proinflammatory cytokines such as TNFα, IL-2, and IL-6, and conversely an increase in the relative proportion of cells that produced IL-10 and TGFβ (Fig. 5A2–H2). In Ly6C^−^ non-classical monocytes/macrophages, a significant treatment-dependent response was observed specifically in the production of proinflammatory cytokines TNFα, IL-2, and IL-6, which were expressed in a comparatively lower proportion of cells in the presence of B cells as compared to saline control (Fig. 5A3–H3). Overall, these results suggested that the observed differential response in cytokine expression within the CD45^hi^ infiltrating population was primarily, though not exclusively, explained by the responses of monocyte/macrophage subsets.

**Fig. 6:**
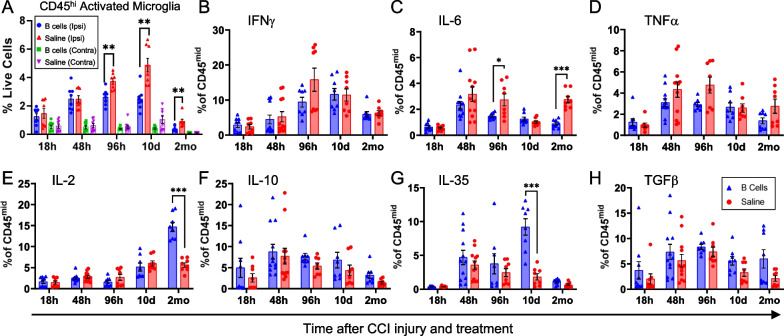
B cell application induces persistent alterations in the activation and inflammatory cytokine profile of resident microglial populations.** A** The numbers of activated microglia were significantly increased in the injured hemisphere at all time points examined, regardless of treatment. B cell application at the time of injury significantly and persistently reduced the proportion of activated pro-inflammatory microglia starting at 4 days and up to 2 months post-injury. **B**–**H** Among the examined cytokines, immunoregulation by B cell treatment on the microglia was less pronounced as compared to that observed in the peripheral immune cells, and mainly included a significant reduction in the proportion of cells expressing high levels of IL-6 **C**, as well as an increase in cells producing IL-2 at 2 months post-injury **E**. Statistical significance was assessed by multiple two-tailed unpaired t-tests for each individual marker and time point and corrected for multiple comparisons using the Holm-Šídák method *p < 0.05; **p < 0.01; ***p < 0.001; ****p < 0.0001

The CD45^mid^ CNS-resident microglia also showed an overall trend towards a less inflammatory functional phenotype in animals treated with B cells at the time of injury. Microglial activation, as indicated by increased CD45 expression [24] was strongly associated with the injured hemisphere (Figs. 2C, 3E, E′, 6A). The proportion of activated microglia was significantly lower after B cell treatment as compared to saline-treated controls starting at 96 h post-CCI and up to 2 months after injury (Fig. 6A). Within the injured hemisphere, a significantly lower percentage of the microglia produced the inflammatory cytokine IL-6 after B cell treatment (Fig. 6C). Conversely, the presence of B cells at the time of injury was associated with a significant increase in the proportion of microglia that produced the regulatory cytokine IL-35 at 10 days post-CCI and IL-2 at 2 months post-injury (Fig. 2E, G).

### Infiltrating CD45^hi^ monocytes/macrophages are necessary mediators of B cell-associated functional neuroprotection after CCI

**Fig. 7:**
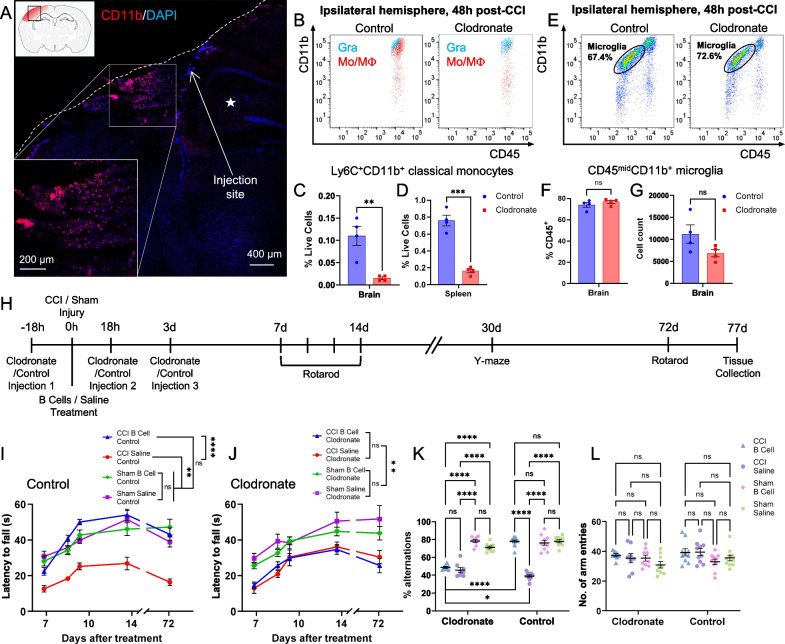
The neuroprotective effect of B cells in TBI requires infiltrating peripheral monocytes/ macrophages at the site of injury. **A** Overview of the CCI injury site at 18 h, showing abundant infiltration of CD11b^+^ monocytes (*red*) throughout the region. The *dashed line* indicates the pial surface of the injured brain hemisphere. The *asterisk* indicates the hippocampus. Under the selected imaging conditions, CD11b immunolabeling preferentially identified large infiltrating myeloid cells, rather than resident microglia, which are not apparent, likely due to lower expression of this marker. **B** Intravenous administration of clodronate liposomes 18 h prior to CCI resulted in the specific depletion of CD45^hi^CD11b^+^ infiltrating immune cells in the injured brain hemisphere. No reduction in cell numbers was observed in the infiltrating CD11b^low/−^ lymphocyte population. **C**, **D**. Quantitative analysis confirmed a significant (~ 90%) depletion of Ly6C^+^CD11b^+^ classical monocytes in the brain as well as in the spleen of animals treated with a single dose of clodronate 18 h prior to CCI (*p* < 0.01, two-tailed unpaired t-test; N = 4 animals/ treatment). **E** Clodronate administration did not deplete the resident microglia in the injured hemisphere. **F**, **G** Quantitative analysis confirmed no significant depletion of CD45^mid^CD11b^+^ microglia, either as a proportion of total CD45^+^ immune cells **F**, or in absolute cell counts **G** (*p* < 0.01, two-tailed unpaired *t*-test; N = 4 animals/ treatment). **H** Experimental paradigm for assessing the impact of clodronate-mediated depletion of peripheral myeloid cells on the functional neuroprotective effect of B cell administration. **I, J** Rotarod assessment of motor learning and coordination. In injured animals (*N* = 10/group) that received injections with control liposomes, B cell administration was associated with strong functional neuroprotection, with treated animals performing significantly better than saline-treated controls, and indistinguishable from sham-injured controls (*p* < 0.001 interaction, two-way repeated-measures ANOVA followed by Tukey's multiple comparisons test). This effect was maintained up to 72 days post-injury **I**. By contrast, in animals treated with clodronate liposomes 18 h before and during the first week post-CCI, the neuroprotective effect of B cell treatment observed in this test was mostly abolished **J**. **K**, **L** Y-maze assessment of short-term spatial learning. **K** The neuroprotective effect of B cell treatment was lost in injured animals treated with clodronate, which performed similar to saline-treated injured controls and significantly worse than sham-injured controls. In the control condition, when myeloid cells were not depleted, injured B cell-treated animals performed significantly better than saline-treated injured controls and similarly to sham-injured animals (*p* < 0.0001, three-way ANOVA, followed by Tukey's multiple comparisons test). **L** No difference was observed in the number of total arm entries between any of the injured and sham groups, indicating no bias in total motor and exploratory activity between treatment groups. *p < 0.05; **p < 0.01; ***p < 0.001; ****p < 0.0001

CD45^hi^ CD11b^+^ monocytes/macrophages infiltrate specifically and abundantly at the injury site within 18 h after CCI and interact directly with the exogenous B cells applied in situ (Fig. 7A). Because of their prominence—comprising over 60% of the infiltrating peripheral immune cells—and their persistence for up to 2 months in situ, we hypothesized that monocytes/macrophages may be key mediators of the neuroprotective immunomodulatory effects observed after B cell treatment. We used intravenous administration of clodronate liposomes to specifically deplete highly phagocytic CD45^hi^ CD11b^hi^ monocytes/macrophages, and confirmed that the systemic depletion translated into a highly significant reduction of approximately 90% in the number of monocytes/macrophages infiltrating the brain at 48 h after CCI (Fig. 7B, C). To effectively decouple the interaction of infiltrating monocyte/macrophage with the exogenous B cells, clodronate liposomes were administered the day before CCI injury and at 18 and 72 h post-CCI (Fig. 7H), ensuring depletion of monocytes/macrophages during the first 7–10 days post-CCI. Behavioral assessments using tests in which we previously found strong functional neuroprotection associated with B cell treatment [8, 9] were used to determine the impact of monocyte/macrophage depletion at the time of injury. In the rotarod test, which assesses both coordination and motor learning over multiple trials, B cell administration in control animals was reproducibly associated with strong functional neuroprotection, with treated animals performing significantly better than saline-treated controls, and indistinguishable from sham-injured controls (Fig. 7I). Notably, this effect was maintained up to 72 days post-injury, long past the survival time of the exogenous B cells. By contrast, in animals that were depleted of monocytes/macrophages by treatment with clodronate liposomes, the neuroprotective effect of B cell treatment was mostly abolished (Fig. 7J). Monocyte/macrophage-depleted animals showed a strong effect of injury on the rotarod test, and B cell treatment did not rescue performance (Fig. 7J). Similarly, in a functionally separate paradigm, the Y-maze assessment of short-term spatial learning, the neuroprotective effect of B cell treatment was lost in animals treated with clodronate liposomes, which performed similarly to saline-treated controls and significantly worse than sham-injured controls (Fig. 7K, clodronate). When the monocyte/macrophage compartment remained intact, B cell-treated animals performed significantly better than saline-treated CCI injured animals and similarly to the sham-injured groups (Fig. 7K, control). Differences in performance in the Y-maze assessment could not be attributed to motor or exploratory deficits, since no difference was observed in the number of total arm entries between any of the injured or sham groups (Fig. 7L).

### Reciprocal regulatory modulation between exogenously administered B cells and infiltrating peripheral monocytes/macrophages changes the default inflammatory response in the injury microenvironment

**Fig. 8:**
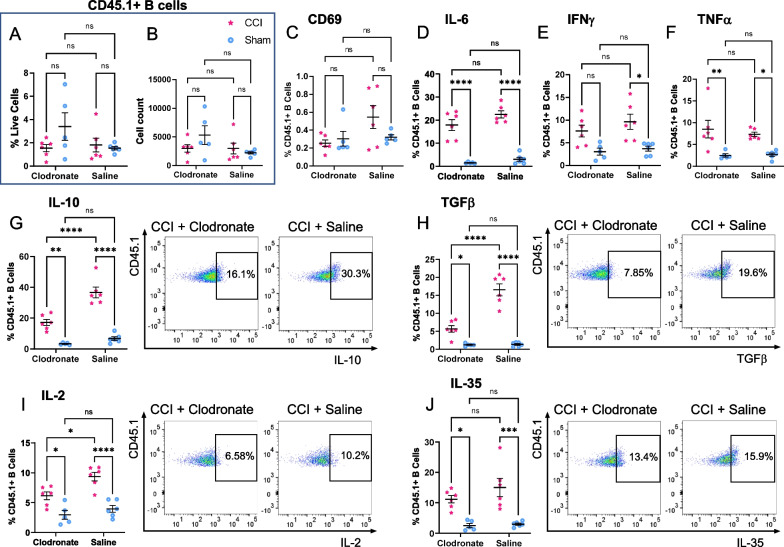
Infiltrating peripheral monocytes/ macrophages at the site of injury are required for specific regulatory B cell activation. Circulating monocytes were depleted with clodronate liposomes 24 h prior to CCI/sham-injury (*N* = 6/group). Control animals received saline liposomes 24 h prior to CCI/sham-injury (*N* = 6/group). B cells were injected intraparenchymally at the time of injury and retrieved after 48 h in situ. **A**, **B** Clodronate administration did not affect the survival of B cells in situ. **C**–**J** Flow cytometry analysis of cytokine expression in the exogenous B cells showed a significant impact of monocyte/macrophage depletion on the proportion of B cells positive for IL-10 **G**, TGFβ **H**, and IL-2 **I**. For most other cytokines and activation markers, there was a significant injury-associated response, however this was independent of myeloid cell depletion. Statistical significance was assessed by two-way ANOVA, followed by Šídák’s multiple comparisons test. *p < 0.05; **p < 0.01; ***p < 0.001; ****p < 0.0001

We next asked whether the presence of peripheral infiltrating monocytes/macrophages is necessary for activation and regulatory cytokine production in the transplanted exogenous B cells. Mice were treated with clodronate liposomes or control saline liposomes 24 h before CCI or sham injury (*N* = 6 per condition and per treatment), and all received an intraparenchymal injection of 10 × 10^6^ B cells from WT C57Bl6 donors. The exogenous B cells were retrieved 48 h after injection into the lesioned or sham-lesioned hemisphere, at a time point when the strongest B cell activation and cytokine response was observed (Fig. 1). Clodronate administration did not impact B cell survival, as indicated by the similar numbers of B cells retrieved under all experimental conditions (Fig. 8A, B). As previously, analysis of the cytokine response in the exogenous B cells was performed after 4 h of exposure to brefeldin A in vivo. Results confirmed an overall strong response of B cells to the CCI microenvironment at this time point, with significantly higher proportions of the B cell population expressing key pro- and anti-inflammatory cytokines in samples from the injured as compared to sham-injured hemispheres (Fig. 8). When the effect of monocyte/macrophage depletion was considered, significant differences were restricted to a subset of cytokines. In animals that received clodronate, a significantly lower proportion of the exogenous B cells produced the regulatory cytokines IL-2, IL-10 and TGFβ (Fig. 8B, D, E), although no significant differences were observed in the production of pro-inflammatory cytokines. This result suggests that the presence of peripheral infiltrating monocytes/macrophages is not required for overall B cell activation in situ, but it is necessary for the specific induction of a regulatory phenotype in the exogenous B cells.

**Fig. 9:**
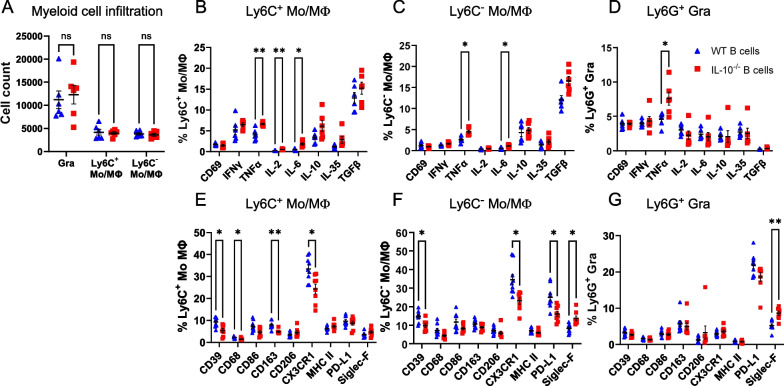
B cell-derived IL-10 is a key regulator of pro-inflammatory responses in infiltrating monocytes/macrophages at the site of injury. **A** Animals treated with either WT or IL-10^−/−^ B cells (N = 6/group) and subjected to CCI showed no difference in the overall numbers of infiltrating myeloid immune cells in the ipsilateral hemisphere 48 h after injury. **B** Assessment of the cytokine response in Ly6C^+^CD11b^+^ classical monocytes showed a significantly higher proportion of cells that were positive for the pro-inflammatory markers TNFα, IL-2, and IL-6 in the absence of IL-10 in the exogenous B cells. **C** Similarly, in Ly6C^−^CD11b^+^ non-classical monocytes, the proportion of cells positive for TNFα and IL-6, but not other cytokines, was significantly increased in animals treated with IL-10^−/−^ B cells. **D** Among infiltrating neutrophils, TNFα-expressing cells were proportionally enriched in CCI injured hemispheres that received IL-10^−/−^ B cells. **E** In animals treated with IL-10^−/−^ B cells (N = 8–9/group) and subjected to CCI, phenotypic changes in infiltrating Ly6C^+^CD11b^+^ classical monocytes included a significant reduction in regulatory markers CD163, CD39, and CX3CR1, but also in the activation marker CD68. **F** In Ly6C^−^CD11b^+^ non-classical monocytes, the proportion of cells positive for CD39, CX3CR1, and PD-L1 decreased while Siglec F increased significantly. **G** The proportion of Siglec F positive cells among infiltrating neutrophils increased significantly when IL-10^−/−^ B cells were applied. Statistical significance was assessed by two-way ANOVA and multiple two-tailed unpaired t-tests for each individual marker and corrected for multiple comparisons using the Holm-Šídák method. *p < 0.05; **p < 0.01; ***p < 0.001; ****p < 0.0001

In turn, regulatory B cells are likely to alter the response of infiltrating myeloid cells after CCI. We investigated the effect of IL-10, one of the key anti-inflammatory cytokines produced by B cells in this context. When IL-10^−/−^ B cells were injected at the lesion site, and infiltrating myeloid cells were examined 48 h after injury, we found that the pro-inflammatory cytokine TNFα was produced in a significantly larger proportion of Ly6C^+^ and Ly6C^−^ monocytes/macrophages, as well as in infiltrating granulocytes (Fig. 9). The percentage of Ly6C^+^ monocytes/macrophages that produced IL-6 and IL-2 was also significantly higher in animals that received IL-10^−/−^ B cells, while in Ly6C^−^ monocytes/macrophages only the percentage of IL-6^+^ cells was significantly increased (Fig. 9). Other, regulatory, cytokines did not show statistically significant differences associated with the type of B cells used. Interestingly, certain surface markers which are associated with a regulatory phenotype, such as CD163 in Ly6C^+^ monocytes/macrophages and PD-L1 in Ly6C^−^ monocytes/macrophages were significantly reduced in the presence of IL-10^−/−^ B cells as compared to WT controls (Fig. 9E, F).

Together, these results suggest that the presence of inflammatory infiltrating monocytes/macrophages induces a regulatory response in B cells, which subsequently contribute to a downregulation of pro-inflammatory phenotype and neurotoxic cytokines in the myeloid cells infiltrating the brain injury site.

## Discussion

Recent research has significantly advanced our understanding of the non-canonical role of B cells in tissue injury and of their complex responses that modulate neuroinflammation [9, 11–13]. In response to injured, inflammatory environments, B cells have been shown to adopt a regulatory phenotype capable of immunosuppression and immunomodulation [25]. Several recent studies have focused on the regulatory and pro-regenerative effect that B cells have in the context of tissue injury in the CNS and in other tissues [8, 9, 12, 26–30]. The use of B cells as a versatile, multimodal immunomodulatory therapeutic may constitute a promising approach in the context of highly complex pathologies such as TBI.

**Fig. 10:**
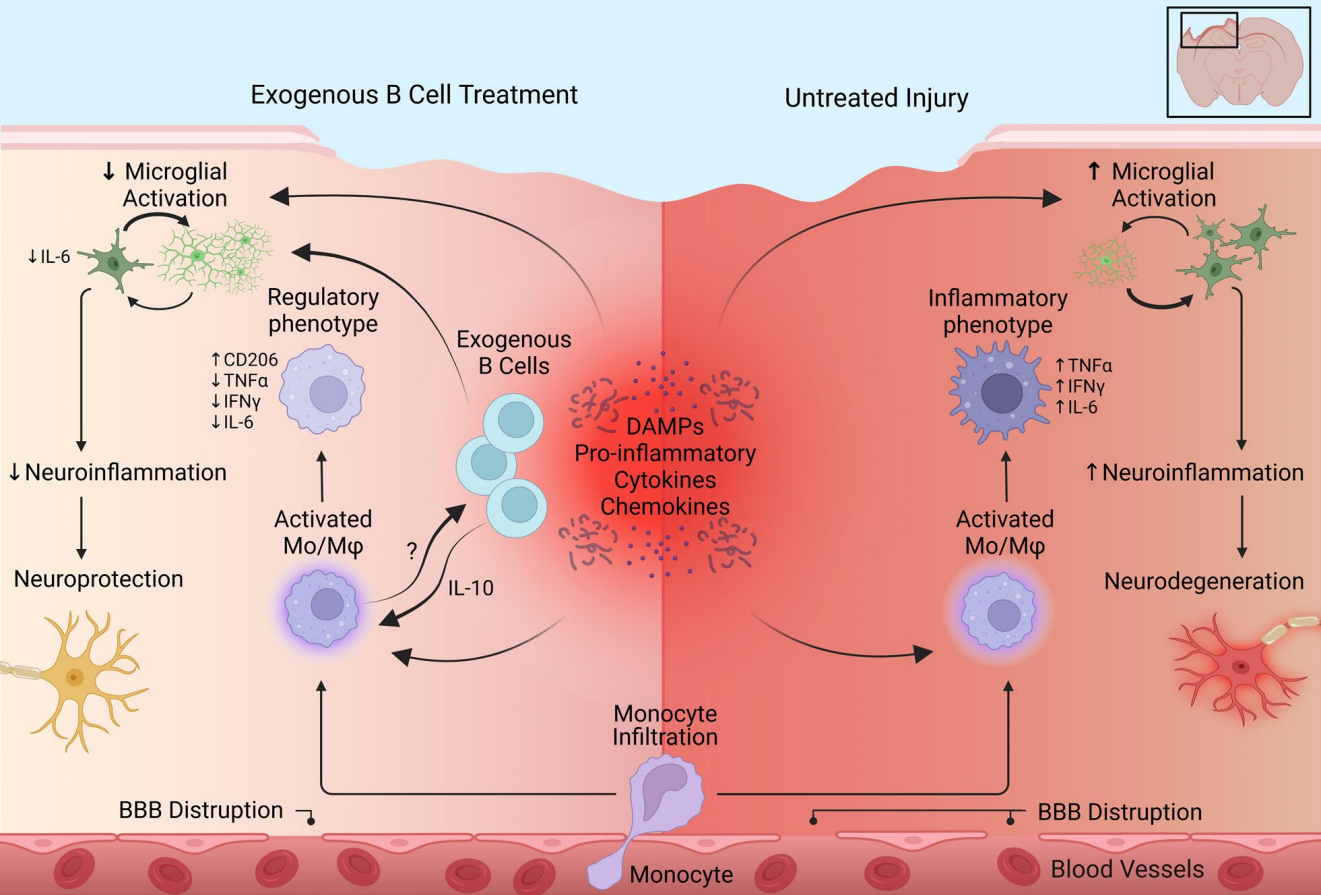
Summary of emerging mechanism of action underlying the observed indirect neuroprotective effects of exogenous B cells in the context of acute and chronic brain injury in the current study. Exogenous B cells injected directly into the injury site receive activating signals from the injured microenvironment as well as from infiltrating immune cells and acquire a regulatory phenotype. The activated exogenous B cells show a reciprocal functional interaction with infiltrating and resident myeloid cells, including monocytes/macrophages and microglia throughout the lesion site, reducing the baseline inflammatory response in these immune populations

The present study sets out to investigate some of the cellular interactions that enable the previously observed neuroprotective effects of exogenous B cells in the context of contusion TBI [8]. Recent work using proteomic analysis in skin wound healing indicated that B cells introduced directly into an injured tissue initiate highly complex responses and alter numerous inflammatory and regenerative processes to accelerate tissue healing—a phenomenon we termed *pligodraxis* [9]. We have therefore initiated our analysis by focusing on the in situ interactions between the experimentally introduced B cells and the most abundant subsets of immune cells that play a key role in the response to TBI. We found that the exogenous B cells act as a dynamic immunoregulator of infiltrating monocytes/macrophages in the injured tissue, shifting their normally inflammatory response to injury towards a milder, regulatory and pro-regenerative phenotype (Fig. 10). We propose that these interactions are likely initiated by damage-associated molecular factors released in the injury microenvironment [9]. Some of these molecules, such as heat-shock protein 60 (HSP60) and high mobility group box 1 (HMGB1) protein have been shown to activate B cells via Toll-like receptors, leading to pleiotropic cytokine production [9]. It is likely that the same damage-associated endogenous molecules stimulate the infiltrating immune cells, and during the normal immune response after CCI, induce the abundant secretion of cytotoxic inflammatory mediators including reactive oxygen species (ROS), TNFα, and IL-6 [1, 2].

Myeloid innate immune cells—granulocytes and monocyte/macrophages—represent the majority of the inflammatory and immune cells to first cross the blood–brain barrier permeabilized by the injury [1]. These cell subsets are likely to have a disproportionate impact on the injured CNS microenvironment and are also the first to encounter and interact with the exogenous B cells injected into the injury site. Indeed, we found that infiltrating CD11b^+^ monocytes/macrophages distribute throughout the injured tissue as soon as 18 h after CCI and appear to interact closely with the B cells in situ (Fig. 7A). Although conflicting roles have been ascribed to monocytes/macrophages that arrive at the site of CNS injury because of their multifunctional potential, overall, detrimental effects associated with inflammation in this context appear to dominate [31–35]. After TBI, monocytes that invade the brain parenchyma have a negative impact on neuronal survival and functional recovery [15], and in spinal cord injury models direct contact between macrophages and neurons was correlated with axon retraction [36]. Experimental depletion of macrophages and microglia may even be beneficial for recovery of locomotor function after spinal cord injury, at least in the early stages after injury [37]. In the present study, we observed no strong effect of peripheral monocyte depletion on the functional recovery of mice after CCI in the rotarod and Y-maze assays (Fig. 7G, H). Control saline-treated animals subjected to peripheral monocyte depletion using clodronate liposomes were not statistically different from individuals with intact peripheral immunity. This effect suggests that under baseline conditions, pro- and anti-inflammatory responses in infiltrating monocytes/macrophages may balance under the complex in situ stimulation by TLR ligands [38–40].

The current study shows that this default response can be significantly and robustly modulated by the experimental addition of B cells in the injury site. In the presence of B cells, infiltrating myeloid cells overall were significantly less likely to produce TNFα, IFNγ, and IL-6, and significantly more likely to generate regulatory factors such as IL-10, IL-35, and TGFβ instead. While a detailed examination of all infiltrating immune subsets was outside the scope of this investigation, we found that Ly6C^+^ and Ly6C^−^ monocytes/macrophages dominate the immune infiltrate that survives at chronic time points after injury. Moreover, these myeloid subsets show the most complex differential responses in cytokine expression associated with B cell treatment in the injured microenvironment. Supporting this notion, among Ly6C^+^ classical monocytes/macrophages, a significantly higher proportion expressed CD206, a marker of alternatively activated, regulatory macrophages [23].

Since microglia, the resident immune cells of the CNS, have a shared myeloid origin with monocytes through the embryonic yolk sac precursors [41], we hypothesized that at least some of the mechanisms involved in the B cell–monocyte/macrophage interactions may translate to the resident microglial populations. Indeed, we found that the proportion of highly activated microglia, as indicated by elevated expression of CD45 [24], was significantly reduced in the presence of exogenous B cells, and this effect persisted into the chronic phase post-injury, when the applied B cells were no longer present. The long-lasting effect of B cell treatment at 2 months post-CCI included a reduction in cells producing IL-6 and a significant increase in IL-2, which, although typically associated with lymphocytes, can be produced by a variety of myeloid cells, including microglia [42, 43]. Together these results underscore the therapeutic potential of the propagating effect of the initial acute B cell therapy, which may modulate outcomes far beyond the time frame of their direct cell–cell interactions.

Importantly, the present study illustrates a novel bi-directional association between the therapeutic exogenous B cells and the endogenous infiltrating monocyte/macrophage populations in the context of cell therapy in vivo. The presence of peripheral infiltrating monocytes/macrophages was required for the activation and secretion of regulatory cytokines in the exogenous B cells. In turn, at the same time point after injury, B cell-derived IL-10 was required for anti-inflammatory biasing of infiltrating Ly6C^+^ monocytes/macrophages, reducing their production of TNFα and IL-6. While IL-10 is known to downregulate macrophage gene expression [23], these data confirm that the administered therapeutic B cells produced a sufficient amount of this cytokine to achieve measurable immunomodulation in the local cellular microenvironment. IL-10 also activates STAT3-mediated expression of genes associated with an M2-like phenotype [39], however in the present study we primarily observed a decrease in inflammatory cytokines in this cell subset, rather than an upregulation of anti-inflammatory markers. Since the response of B cells in situ is pleiotropic, it will be important to examine in future studies the factors and pathways involved in the upregulation of anti-inflammatory cytokines, including IL-10 itself, IL-35, TGFβ, and others, which are also induced by the exogenous therapeutic B cells. Moreover, while the present study has focused on one of the cellular mechanisms through which B cells modulate the injury microenvironment after CCI, this investigation is certainly not exhaustive—additional infiltrating immune populations, such as neutrophils and T cells may also play a role and interact with the exogenous B cells. It is likely that the reciprocal interactions between the infiltrating myeloid cells and the exogenous B cells are mediated by a multifactorial interplay of cytokines and surface receptors and potentially other inflammatory and immune cell types at the site of injury. B cells are increasingly recognized for their versatility in adopting regulatory phenotypes, which have been described in a variety of B cell subsets, including transitional B cells, marginal-zone B cells, transitional-2 marginal-zone precursor B cells, B1 cells, plasmablasts, and plasma cells [44]. While the present study used a mixed B cell population resulting from splenic isolation, which includes most of these subtypes [12], it will be of interest to examine the response effectiveness in each of these subsets independently. In addition, since the effects described here are characterized only in male animals, it will be important to replicate these investigations in females, in which certain cellular responses to TBI may be differently regulated [45].

To our knowledge, the results of the present study provide a first insight into the cellular responses of mature naïve splenic B cells in CNS injury, as well as some of the key cell–cell interactions that mediate their complex immunomodulatory and neuroprotective effects in vivo. The reciprocal regulatory interactions with subsets of infiltrating and resident myeloid cells help explain the previously observed long-term effects of acute B cell application, and support the hypothesis that the introduction of B cells into the injured CNS can initiate a regulatory cascade with lasting protective effects. Importantly, this study also confirmed that no detrimental effects are associated with the experimental introduction of relatively large numbers of B cells directly into the brain parenchyma. Together, these data add to the growing evidence that purified B cells are powerful regulatory cells in the context of tissue injury and may represent a promising option for the development of safe and effective cell therapies for the treatment of traumatic brain injuries.

## Supplementary Information

**Additional file 1: Figure S1.** Time-dependent response of exogenous mature naive B cells retrieved from the injured brain. Representative examples of biaxial plots illustrate the typical cell numbers and distribution indicating changes in cytokine expression patterns in the exogenous B cells after exposure to the CCI microenvironment for variable amount of time. Note that the gates are illustrated for orientation and comparison between timepoints. Precise quantitative gating was performed using controls within each examined timepoint.

## Data Availability

All datasets used and/or analyzed during the current study are available on request from the corresponding author.

## Abbreviations

ANOVA: Analysis of variance
BBB: Blood–brain barrier
CCI: Controlled cortical impact
CD: Cluster of differentiation
CNS: Central nervous system
CX3CR1: CX3C motif chemokine receptor 1
DAMPs: Damage-associated molecular patterns
DAPI: 4′, 6-Diamidino-2-phenylindoledihydrochloride
EDTA: Ethylenediaminetetraacetic acid
FBS: Fetal bovine serum
HMGB1: High mobility group box 1
HSP60: Heat-shock protein 60
IFNγ: Interferon gamma
IL: Interleukin
MyD88: Myeloid differentiation factor 88
PBS: Phosphate buffered saline
PE: Phycoerythrin
ROS: Reactive oxygen species
STAT3: Signal transducer and activator of transcription 3
TBI: Traumatic brain injury
TGFβ: Transforming growth factor beta
TLR: Toll-like receptor
TNFα: Tumor necrosis factor alpha
tSNE: T-distributed stochastic neighbor embedding
WT: Wild-type
