# Supplementary figures and images for: B cell treatment promotes a neuroprotective microenvironment after traumatic brain injury through reciprocal immunomodulation with infiltrating peripheral myeloid cells
Source: J Neuroinflammation. 2023 May 31;20:133. doi: 10.1186/s12974-023-02812-y (PMC10230748; doi:10.1186/s12974-023-02812-y)

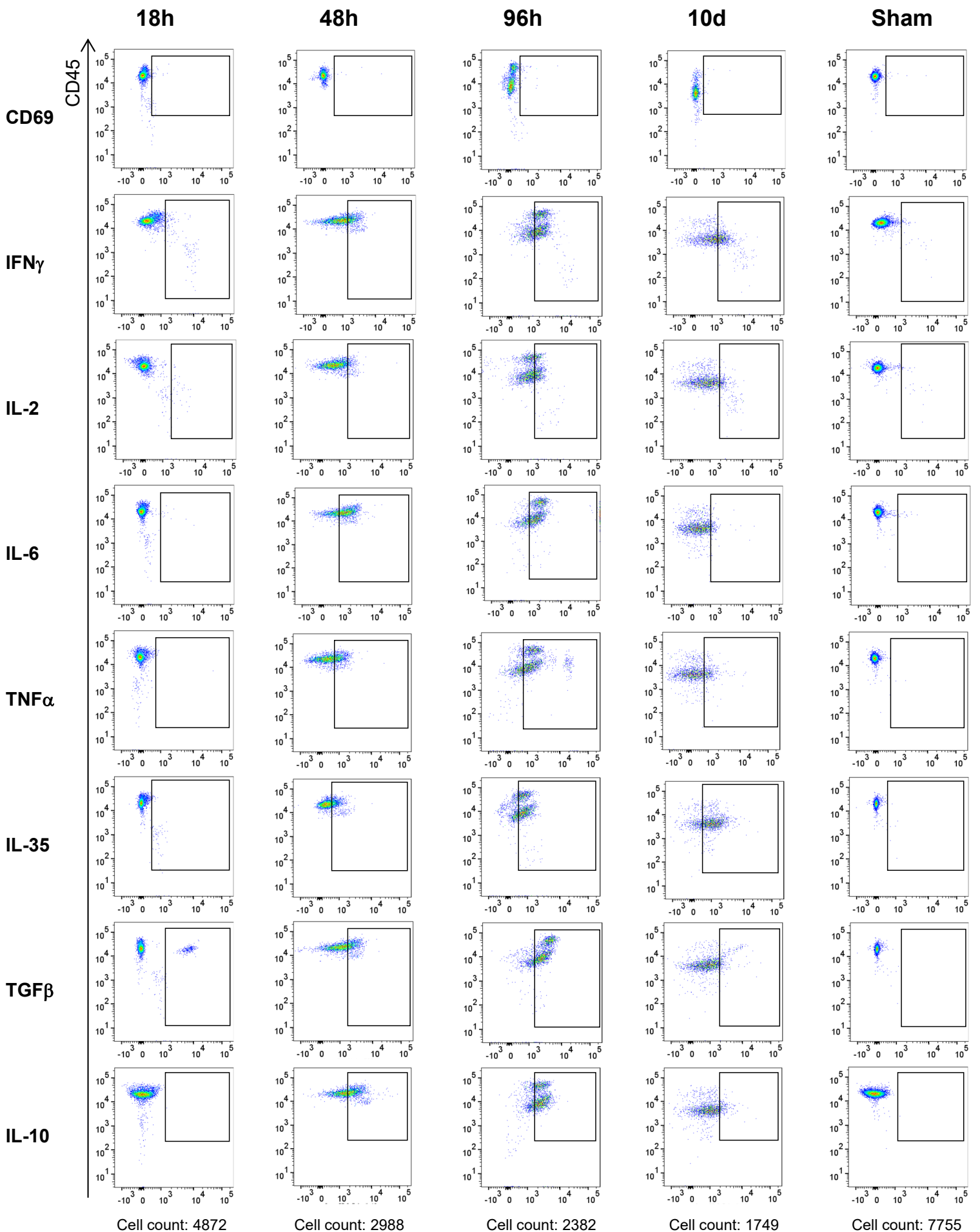

Supplement: Supplementary file 1 — Additional file 1: Figure S1. Time-dependent response of exogenous mature naive B cells retrieved from the injured brain. Representative examples of biaxial plots illustrate the typical cell numbers and distribution indicating changes in cytokine expression patterns in the exogenous B cells after exposure to the CCI microenvironment for variable amount of time. Note that the gates are illustrated for orientation and comparison between timepoints. Precise quantitative gating was performed using controls within each examined timepoint. [file 12974_2023_2812_MOESM1_ESM.pdf]
